# Chitosan-derived N-doped carbon catalysts with a metallic core for the oxidative dehydrogenation of NH–NH bonds†

**DOI:** 10.1039/c9ra08146a

**Published:** 2020-01-02

**Authors:** Priyanka Raju Thombal, Raju S. Thombal, Sung Soo Han

**Affiliations:** School of Chemical Engineering, Yeungnam University 280 Daehak-Ro Gyeongsan Gyeongbuk 38541 Republic of South Korea sshan@yu.ac.kr +82-53-810-4686 +82-53-810-2773

## Abstract

Sustainable metal-encased (Ni–Co/Fe/Cu)@N-doped-C catalysts were prepared from bio-waste and used for the oxidative dehydrogenation reaction. A unique combination of bimetals, *in situ* N doping, and porous carbon surfaces resulted in the formation of the effective “three-in-one” catalysts. These N-doped graphene-like carbon shells with bimetals were synthesized *via* the complexation of metal salts with chitosan and the subsequent pyrolysis at 700 °C. A well-developed thin-layer structure with large lateral dimensions could be obtained by using Ni–Fe as the precursor. Importantly, the Ni–Fe@N-doped-C catalyst was found to be superior for the dehydrogenation of hydrazobenzene under additive/oxidant-free conditions compared to the conventional and other synthesized catalysts. Characterizations by TEM and XPS accompanied by BET analysis revealed that the enhanced catalytic properties of the catalysts arose from their bimetals and could be attributed to the graphitic shell structure and graphitic N species, respectively.

## Abstract 2

Sustainable metal-encased (Ni–Co/Fe/Cu)@N-doped-C catalysts were prepared from bio-waste and used for the oxidative dehydrogenation reaction.

## Graphical abstract

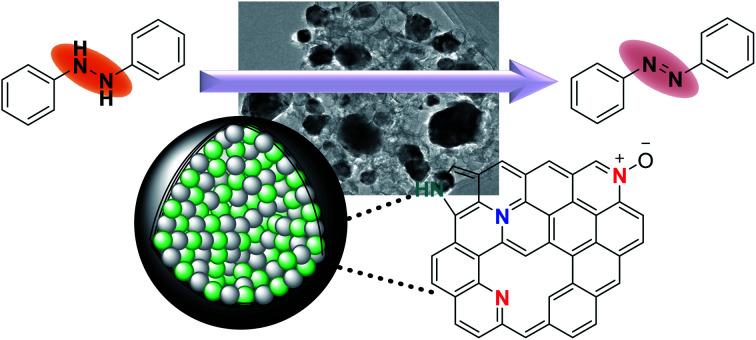

## Introduction

In the past decades, heterogeneous catalysts, including monometallic- and bimetallic-supported catalysts, have been widely utilized for numerous applications. Such catalysts often show distinct electronic and chemical properties from those of their parent metals, which offers the opportunity to obtain new catalysts with enhanced robustness. In 1960, for the first time, bimetallic catalysts started to gain considerable commercial interest for their use in hydrocarbon reforming due to their superior activities, which were unlike those of the monometallic catalysts.^1–3^ These unexpected properties of bimetallic catalysts have inspired many researchers to find suitable applications. Recently, bimetallic catalysts have been widely utilized in many catalytic^4^ and electrocatalytic applications.^5,6^

Heteroatom-doped carbon materials bearing metals have emerged as promising and cost-effective alternatives for promoting organic reactions. These carbon materials can be synthesized by the pyrolysis of glucose, cellulose, β-cyclodextrin, and other biomass-derived supports.^7–11^ Due to their unique properties, such as high graphitized carbon, uniformly doped heteroatoms, and porous structures, such catalysts have been explored for a series of organic transformations including hydrogenation, oxidation, and acid/base-catalyzed reactions.^12–15^ However, the pyrolysis step involved in the preparation of carbocatalysts usually leads to the fusion of carbon nanoparticles, and this resulting aggregation blocks the tunnels of mass transfer and limits active site exposure.^16,17^ Moreover, obtaining well-graphitized carbon with efficient dopants is hard to achieve in practice.^18^ Therefore, it is important to precisely optimize the synergistically active metal sites and N-doped porous structures simultaneously over carbon-based catalysts for better exposure in dehydrogenation reactions.^19–21^

**Scheme 1:**
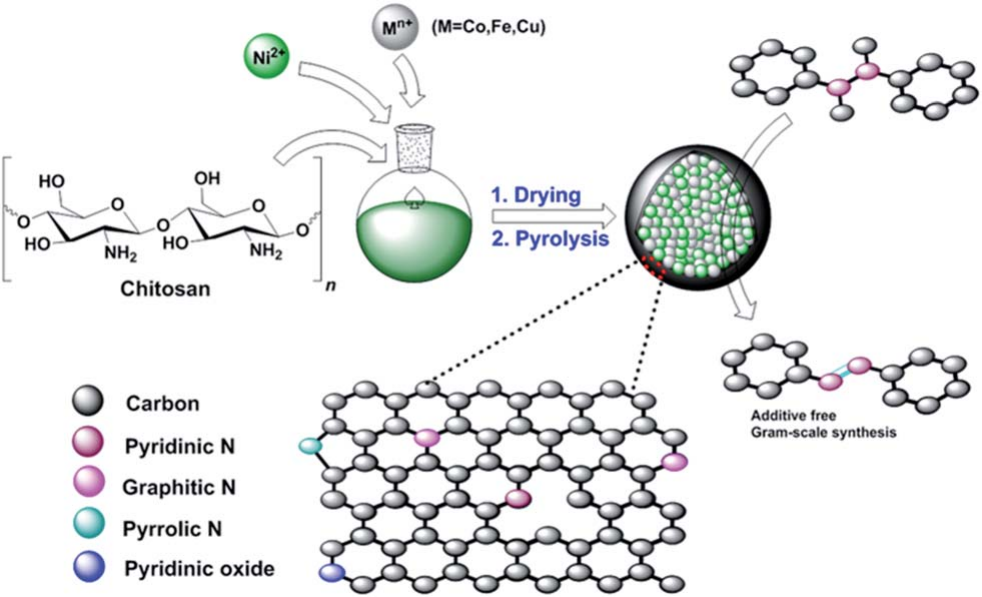
Schematic illustration for the preparation of Ni–M@N-doped-C (M = Co, Fe, Cu) catalysts.

In 2010, Titirici and coworkers described the hydrothermal treatment of chitosan, followed by calcination, for the preparation of carbonaceous materials.^22^ Later, it was shown that chitosan could serve as an excellent precursor to generate N-doped graphene.^23,24^ Importantly, these biomass-derived products are inexpensive, sustainable, biodegradable, and non-toxic. Chitosan is mostly obtained from bio-waste generated from fishery plants, and it includes shrimp or crab shell-derived chitin.^25^ In this regard, the synthesis of ruthenium-cobalt nanoalloys encapsulated in nitrogen-doped graphene from the nanotubes of the Co_3_[Co(CN)_6_]_2_ precursor has been reported by Chen and coworkers.^26^ Based on the aforementioned efforts and our previous work,^27^ we became interested in preparing novel core–shell N-doped carbon materials with bimetallic nanocomposites as the catalyst. Herein, we report a straightforward synthesis of novel (Ni–M)@N-doped-C (M = Co, Fe, Cu) catalysts by a combination of non-precious transition metals with chitosan and subsequent pyrolysis (Scheme 1). Although the preparation of a carbonaceous monometallic cobalt catalyst from chitosan has already been reported,^28^ to the best of our knowledge, the synthesis of nickel-based bimetallic catalysts from chitosan has not been reported. It is noteworthy that due to the synergetic effect of the metal nanocomposites and N-doped carbon coating of these materials, they can be used as efficient bimetallic carbocatalysts in the oxidative dehydrogenation of NH–NH bonds.

## Experimental

### Materials and characterization

Chitosan, hydrazobenzene, NiCl_2_·6H_2_O, CoCl_2_·2H_2_O, FeCl_3_, CuCl_2_·2H_2_O, and solvents were purchased from Sigma Aldrich. All the chemicals were reagent grade and used as received without any purification. The synthesized catalysts were characterized using several extensive techniques. Scanning electron microscopy (SEM) images of all the catalysts were obtained on Hitachi S4600 equipped with an energy dispersive X-ray microanalysis (EDAX) detector. The wide-range X-ray diffraction (XRD) patterns were recorded on Panalytical X'Pert pro MPD using Cu-Kα radiation. The catalysts were scanned from 10 to 90° at a rate of 5° min^−1^. Raman spectra were obtained on a Horiba scientific Raman spectrometer system using a 532 nm wavelength laser. X-ray photoelectron spectroscopy (XPS) was conducted on a Thermo Fisher Scientific K-Alpha system. The specific surface area and the pore-size distributions were determined using Brunauer–Emmett–Teller (BET) nitrogen adsorption–desorption isotherms and the Barrett–Joyner–Halenda (BJH) method, respectively, with a Micromeritics 3-Flex surface characterization analyzer at 77 K. Transmission electron microscopy (TEM) images were acquired on an FEI Tecnai (F20 instrument) high-resolution microscope. The concentrations of the metal species were determined by inductively coupled plasma atomic emission spectrometry (ICP-AES) on a Perkin-Elmer Optima 8300 system.

### Catalyst preparation

The chitosan-based bimetallic materials were synthesized according to the following procedure: in a 500 mL round-bottomed flask provided with a magnetic stir bar, 1.5 gm of chitosan was suspended in 100 mL of water. Next, we added 250 mg (1 : 1) of each NiCl_2_·6H_2_O and metal precursors (Co/Fe/Cu) into the above suspension solution. The pH was maintained around 9 by adding 25% aqueous ammonia solution and the mixture was stirred continuously for 24 h. Hereafter, the obtained solid was repeatedly washed with water to remove excess impurities and reactants. The solid was then separated by a centrifugation process (500 rpm, 5 min) and dried in vacuum at 60 °C overnight. Later, the dried sample was transferred into a tubular furnace for pyrolysis. The furnace was then flushed with argon and the sample was heated to 700 °C at a temperature gradient of 25 °C min^−1^ and the same temperature was held for 2 h. After that, the furnace was cooled down to room temperature. Argon was purged through the furnace constantly during the whole process. The obtained black material was washed with water and ethanol to remove impurities. The catalysts named as Ni–M@N-doped-C (M = Co/Fe/Cu) were obtained after drying the samples at 60 °C in an oven and then storing in a screw-capped vial at room temperature. For the preparation of the monometallic catalysts, the same procedure was followed using single metal precursors.

### Synthesis of azobenzene over the Ni–Fe@N-doped-C catalyst

Typically, calculated amounts of hydrazobenzene (1 mmol) and Ni–Fe@N-doped-C (5 wt%) in ethanol (3 mL) were placed in a 10 mL glass vial, and K_2_CO_3_ (1 mmol) was added to the mixture under an air atmosphere with magnetic stirring to initiate the reaction at 30 °C for 12 h. After the reaction was completed, the catalyst was separated using an external magnet and the crude mass was subjected to column chromatography. After each cycle, the catalyst was isolated from the solution by an external magnet, washed three times with water and acetone, dried under vacuum to remove the residual solvent, and then reused for another reaction cycle.

## Results and discussion

### Properties of the Ni–M@N-doped-C catalysts

**Fig. 1:**
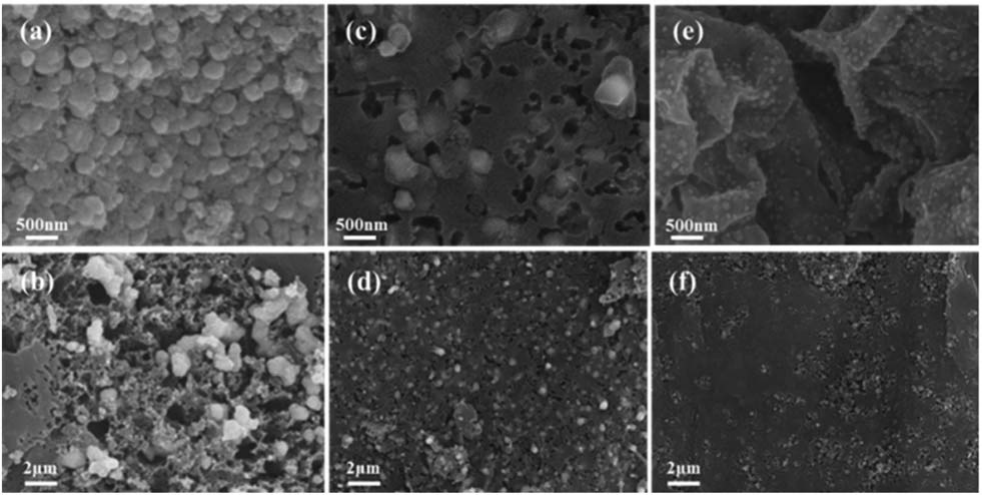
FE-SEM images of the CS-bimetallic nanocomposites: Ni–Co@N-doped-C (a and b), Ni–Fe@N-doped-C (c and d), and Ni–Cu@N-doped-C (e and f).

The surface morphology of the chitosan-derived bimetallic catalysts was initially probed by SEM. Fig. 1 exhibits the typical SEM images of the Ni–Co@N-doped-C, Ni–Fe@N-doped-C, and Ni–Cu@N-doped-C catalysts with different scale bars. The images show that the catalysts have an irregular particle-like structure and metal nanocomposites are equally embedded in the carbon structure. The core–shell morphology is clearly distinct in all the catalysts. The EDS results are shown in Fig. S1(a–c),† which provide the elemental distributions of the catalysts. A uniformly dispersed nature of both metals (Ni and Co/Cu/Fe) in all three pyrolyzed catalysts was observed.

**Fig. 2:**
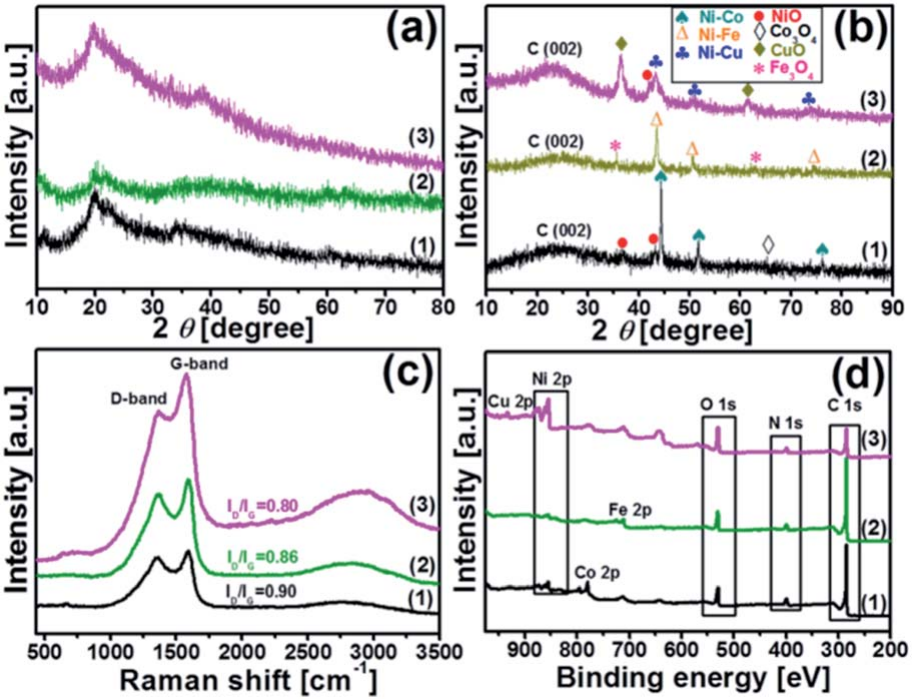
Wide-angle XRD patterns of (a) before pyrolysis; (b) after pyrolysis; (c) Raman spectra and (d) XPS survey spectra of the catalysts: (1) Ni–Co@N-doped-C, (2) Ni–Fe@N-doped-C, and (3) Ni–Cu@N-doped-C, respectively.

XRD analysis was carried out to understand the structure of the synthesized catalysts. Fig. 2(a and b) illustrate the XRD patterns of the chitosan-bimetallic catalysts before and after pyrolysis. Before pyrolysis, the XRD patterns exhibit a broad characteristic peak at 2*θ* = 20°, which belongs to chitosan. Peaks pertaining to the metal nanocomposites were not clearly observed in these patterns, which was possibly due to the low concentration of metals.^29^ After pyrolysis, the peak observed for chitosan at 2*θ* = 20° became weak, resulting in a new diffraction peak at 18–30° corresponding to the (002) plane of graphitic carbon.^30^ This transition of peaks suggests the decomposition of chitosan along with the conversion of carbon-rich biomass into graphitic carbon nanostructures in all the catalysts. Since nickel, copper, cobalt, and iron are neighbors in the periodic table, they have very close lattice parameters and apparently close standard identification peaks in the XRD database.^31^ The Ni–Co@N-doped-C catalyst shows diffraction peaks at 44.40°, 51.67°, and 76.08°; these are ascribed to the face-centered cubic structure of the Ni–Co alloy (PDF#01-074-5694), which can be indexed to the (111), (200), and (220) crystalline reflections, respectively. Additionally, the peaks with very less intensity observed at 37.10° and 43.30° can be ascribed to the NiO phase (PDF#00-047-1049) and the peak at 64.70° is ascribed to the Co_3_O_4_ phase (PDF#01-073-1701). The Ni–Fe@N-doped-C catalyst shows peaks at the 2*θ* values of 43.30°, 50.58°, and 74.45°, which are assigned to the face-centered cubic and monoclinic phases of the Ni–Fe crystallographic structure (PDF# 00-023-0297). On the other hand, the diffraction peaks at 35.63° and 62.88° can be indexed to the cubic phase crystallographic structure of Fe_3_O_4_ (PDF# 01-086-1344). As for the bimetallic catalyst Ni–Cu@N-doped-C, the diffraction peaks at 2*θ* of 43.46°, 50.58°, and 74.30° indicate the presence of a face-centered cubic structure of the Ni–Cu alloy (PDF# 00-047-1406). Along with this, the peaks at 36.38° and 61.52° confirm the CuO (PDF# 01-073-6023) phase, whereas a peak at 42.03° is obtained due to the NiO phase.^32,33^ The peaks observed in the XRD patterns confirmed the presence of metals/metal oxides in the synthesized monometallic Ni@N-doped-C and Fe@N-doped-C catalysts (for further details, see Fig. S2†).

Fig. 2c shows a typical Raman spectrum, which can be used to probe the graphitic carbon formation in the catalysts. All the three spectra of the synthesized catalysts consist of two prominent peaks at ∼1356 cm^−1^ for the D band and 1592 cm^−1^ for the G band, along with a wide band extending from about 2900 cm^−1^ due to the combination modes of the D and G bands. The D band indicates a defect-induced non-perfect crystalline structure, while the G band shows an in-plane vibration of the sp^2^ carbon atoms.^34^ The values of the intensity ratio *I*_D_/*I*_G_ of different catalysts followed the sequence Ni–Co@N-doped-C (0.90) > Ni–Fe@N-doped-C (0.86) > Ni–Cu@N-doped-C (0.80). Herein, the first two catalysts presented a relatively high *I*_D_/*I*_G_ value; this indicates the presence of abundant defects in the graphitic network of the catalyst, which can mostly affect the catalytic performance.

**Fig. 3:**
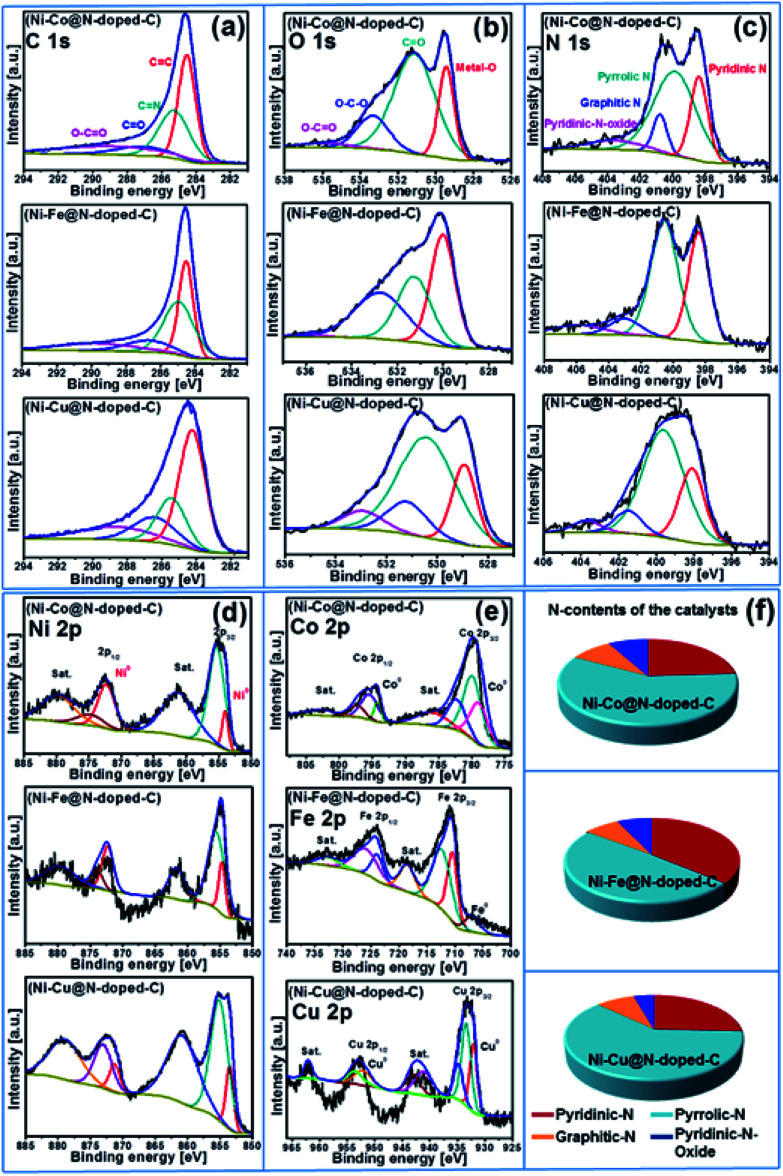
XPS spectra of (a) C 1s, (b) O 1s, (c) N 1s (d) Ni 2p, (e) Co 2p/Fe 2p/Cu 2p for the Ni–Co@N-doped-C, Ni–Fe@N-doped-C, and Ni–Cu@N-doped-C catalysts; (f) nitrogen contents of the catalysts.

X-ray photoelectron spectroscopy (XPS) was carried out to determine the surface valence states of the characteristic elements in the synthesized bimetallic catalysts (Fig. 2d). The high-resolution XPS scans of the C 1s, O 1s, N 1s, Ni 2p, and Co 2p/Fe 2p/Cu 2p regions with curve-fitting spectra for the synthesized Ni–Co@N-doped-C, Ni–Fe@N-doped-C, and Ni–Cu@N-doped-C catalysts are illustrated in Fig. 3. The deconvoluted C 1s spectrum (Fig. 3a) of the three catalysts contains four peaks at different B.E. values. The C 1s peaks at B.E. of 283.5–284.5, 284.7–285.2, 286.1–287.0 and 288.6–289.7 eV are assigned to carbon atoms in the forms of C=C, C=N, C–O/C–N, and O–C=O, respectively. The eminent peaks at B.E. of 283.5–284.5 eV indicate that most carbons in the bimetallic catalysts are aromatic, which coincides with the graphitic structure observed in the XRD patterns (Fig. 2b). The O 1s spectra of these three catalysts are shown in Fig. 3b and all of them contain four peaks regarding the various O functionalities; among these, the peaks at B.E. of 528.9–530.0 eV correspond to the lattice oxygen involved in the metal framework, such as Ni–O or Co–O/Fe–O/Cu–O bonds. The peaks at B.E. of 530.5–531.2 eV are assigned to C=O, while the peaks at B.E. of 531.1–533.3 eV correspond to the O–C–O bonds. At B.E. of 533.0–535.9 eV, peaks with lower intensities are obtained, which are ascribed to the O–C=O species in the catalysts. The N 1s spectrum (Fig. 3c) of the three catalysts comprises four deconvoluted peaks at different B.E. values. The peaks in the range of 398.0–398.4, 399.6–400.5, 400.7–402 and 401.4–405.8 eV can be attributed to pyridinic N, pyrrolic N, graphitic N, and pyridinic N-oxides, respectively.^35^ It could be clearly observed that pyridinic N and pyrrolic N were the dominant phases in all three catalysts. The second highest intensity was analyzed for the graphitic N moieties, while N-oxides were detected as minor components in all three catalysts. In the Ni 2p spectrum (Fig. 3d) of these three representative catalysts, the deconvoluted peaks at B.E. values in the range of 851.5–858.0 eV are due to Ni 2p_3/2_ and the peaks at 869.7–875.8 eV are due to Ni 2p_1/2_. The peaks located at 853.5–872.2 eV can be ascribed to metallic Ni, whereas the peaks obtained in the range of 861.1–879.7 eV are the satellite peaks of Ni^2+^.^36^ The high-resolution spectra of Co 2p, Fe 2p, and Cu 2p of the Ni–Co@N-doped-C, Ni–Fe@N-doped-C, and Ni–Cu@N-doped-C catalysts are presented in Fig. 3e. In the Co 2p spectra, the characteristic Co 2p_3/2_ and Co 2p_1/2_ peaks associated with spin–orbit splitting are observed. The peaks obtained at the B.E. values of 779.8 and 795.4 eV are ascribed to Co^3+^, while the peaks at 782.2 and 797.2 eV are attributed to Co^2+^. The binding energy of the Co 2p peaks in the range of 778.9–794.2 eV indicates the existence of metallic Co in the catalysts, whereas the satellite peaks of Co^2+^ are located in the range of 785.9–802.7 eV. This study of the Co 2p spectra of the catalyst disclosed the presence of metallic Co, Co^3+^, and Co^2+^ species, which affirmed the results from the XRD study. The deconvoluted spectrum of Fe 2p shows the first peak at 706.8 eV, corresponding to the presence of metallic Fe. Other prominent peaks at the binding energies of 710.3 (Fe 2p_3/2_) and 723.7 (Fe 2p_1/2_) eV account for the presence of Fe^2+^, whereas the peaks at the binding energy values of 712.3 and 725.8 eV are assigned to the Fe^3+^ species. The curve-fitting result of Cu 2p is depicted in Fig. 3e. The Cu 2p_3/2_ and Cu 2p_1/2_ peaks located at 932.0 and 952.1 eV can be due to metallic Cu, while the peak centered at 933.3 eV corresponds to Cu^+^. The binding energies of the peaks observed at 934.7 and 953.4 eV and the shakeup satellites at the B.E. values of 941.0–943.0 and 962.0 eV indicate the presence of the Cu^2+^ species on the catalyst surface.^37–39^ As evidenced by the XPS study, the synthesized catalysts contained metallic Ni–Co/Fe/Cu along with their oxides. The N species contents that play a vital role in the catalytic reactions are shown in Fig. 3f.

The N_2_ adsorption–desorption isotherms, surface area, pore-size distribution, and pore-volume curves of the Ni–Co@N-doped-C, Ni–Fe@N-doped-C, and Ni–Cu@N-doped-C catalysts were probed by BET measurements to substantiate the presence of mesopores in the catalysts (Fig. S3†). All the nanocomposites manifested typical type IV isotherms with an H_3_ hysteresis loop, which confirmed the presence of a mesoporous structure. The BJH pore-size distributions of the catalysts validated the existence of well-developed mesoporous structures of the chitosan-derived bimetallic catalysts. The BET surface area (*S*_BET_), pore volume (*V*_pore_), and average pore diameter (AD_pore_) of the catalysts are shown in Table 1. The values displayed that the Ni–Fe@N-doped-C catalyst possessed the highest BET surface area (176.96 m^2^ g^−1^) and the largest pore volume (0.2441 cm^3^ g^−1^). All three catalysts displayed both narrow pore-size distributions centered at around ∼3.4–3.7 nm and broad pore-size distributions at around >10 nm. However, Ni–Fe@N-doped-C possessed a higher number of small and large pores compared to the other two catalysts, which apparently led to the high surface area and pore volume.^40^

**Table 1:** Physicochemical properties of the catalysts

Catalyst	*S*_BET_ (m^2^ g^−1^)	*V*_pore_ (cm^3^ g^−1^)	AD_pore_ (nm)
Ni–Co@N-doped-C	170.05	0.1629	12.6214
Ni–Fe@N-doped-C	176.96	0.2441	20.0551
Ni–Cu@N-doped-C	8.34	0.0134	10.2482

**Fig. 4:**
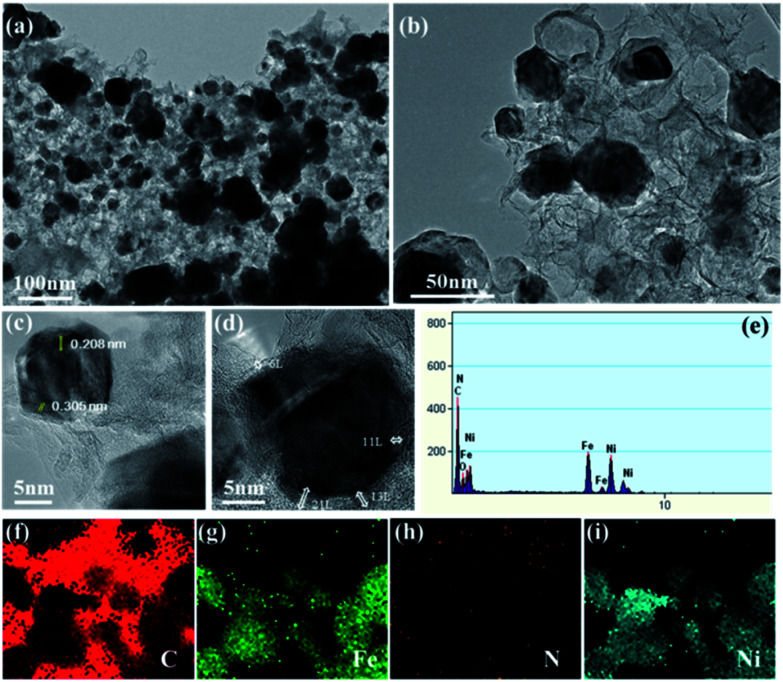
(a and b) TEM images; (c and d) HR-TEM images; (e) EDAX; (f–i) elemental mapping of C, Fe, N, and Ni of the Ni–Fe@N-doped-C catalyst, respectively.

By accumulating the results from SEM and BET, the micro-structure of the synthesized catalyst was characterized by TEM analysis. The TEM and HR-TEM images of the Ni–Fe@N-doped-C catalyst (Fig. 4) show similar results to the morphologies observed in the SEM images (Fig. 1c and d). As revealed in the TEM study (Fig. 4a and b), the Ni–Fe bimetallic nanocomposites with irregular particles with a mean diameter of ∼52 nm were enclosed by N-doped carbon layers. The least number of hollow carbon spheres were also observed in the catalyst originating from the high-temperature carbonization process.^41^Fig. 4c and d show the high-resolution TEM images centered on a single nanocomposite, where the crystal fringes of the core–shell are clearly seen with the *d*-spacings of 0.208 and 0.305 nm; these are in accordance with the (111) and (002) planes of the face-centered cubic (fcc) structure of metals (Ni/Fe) and graphitic carbon material, respectively. Mostly, carbon coatings with layers ∼6–11 along with ∼13–21 thick layers existed, and they were effective sites for the oxidation reaction. The acquired EDAX (Fig. 4e) analysis confirmed the presence of the C, N, O, Ni, and Fe elements in the synthesized nanocomposites. Besides, the images of elemental mapping from TEM show that C, Fe, N, and Ni (Fig. 4f–i) are homogenously distributed on the catalyst. From the inductively coupled plasma atomic emission spectrometry (ICP-AES) analysis of the Ni–Fe@N-doped-C catalyst, the concentrations of the Ni and Fe metals were found to be 12.99 wt% and 18.41 wt%, respectively.

### Catalytic activity of the synthesized bimetallic catalysts ((Ni–Co/Fe/Cu)@N-doped-C)

The synthesized bimetallic catalysts were tested for the oxidative dehydrogenation of hydrazobenzene to azobenzene (Table 2). To obtain the best optimized conditions, the reactions of hydrazobenzene were carried out in the presence of the three synthesized catalysts (10 wt%) in EtOH at room temperature under an open-air atmosphere (entries 1–3). The Ni–Fe@N-doped-C catalyst provided the highest yield (67%) over the other Ni–Cu@N-doped-C and Ni–Co@N-doped-C catalysts possibly due to its greater surface area and good pore-size distribution. On increasing the reaction temperature to 80 °C, the yield of azobenzene did not improve, and 20% of hydrazobenzene remained unreacted (entry 4). Importantly, decreasing the amount of Ni–Fe@N-doped-C to 5 wt% sufficiently produced a higher yield (entry 6, 69%). On the other hand, upon increasing the amount of the catalyst to 20 wt%, azobenzene was produced in a slightly lower yield (entry 5). The reactions using the monometallic Ni@N-doped-C and Fe@N-doped-C catalysts did not improve the yield of azobenzene (entries 7–8). Moreover, the reaction using 10 wt% of commercially available chitosan (CS) did not produce the azobenzene product at all due to the absence of either carbon surfaces or metal ions, which are responsible for the dehydrogenation reaction (entry 9). Additionally, reactions with other carbon-based catalysts were performed to get better insights into the reaction parameters (entries 10–12). The reaction in the presence of graphite flakes, activated carbon, and carbon nanotubes (CNT) produced azobenzene in lower yields, suggesting an important role of the metal ions in this reaction.

**Table 2:** Catalytic oxidation of hydrazobenzene over different catalysts^a^

Entry	Catalyst	Temp [°C]	Recovered hydrazobenzene [%]	Azobenzene yield [%]
1	Ni–Co@N-doped-C	30	40	51
2	Ni–Cu@N-doped-C	30	38	49
3	Ni–Fe@N-doped-C	30	25	67
4	Ni–Fe@N-doped-C	80	20	63
5^b^	Ni–Fe@N-doped-C	30	15	66
6^c^	Ni–Fe@N-doped-C	30	19	69
7^c^	Ni@N-doped-C	30	39	41
8^c^	Fe@N-doped-C	30	45	33
9	CS	30	99	00
10	Flake graphite	30	51	31
11	Activated carbon	30	45	46
12	CNT	30	67	30

a Reaction conditions: 1 mmol hydrazobenzene, 10 wt% catalyst, 3 mL EtOH, 30 °C for 12 h under open air.

b 20 wt% of catalyst was used.

c 5 wt% of catalyst was used. CS = chitosan, CNT = carbon nanotubes.

Furthermore, the effects of solvents and bases were studied using 5 wt% Ni–Fe@N-doped-C at room temperature (Table 3). The reaction of hydrazobenzene in different solvents produced azobenzene in 15–50% yields. Among these, PEG-400, ethylene glycol, THF, and *t*-BuOH were better solvents (entries 3–4, 6–7). However, azobenzene was produced in lower yields when other solvents such as γ-valerolactone (GVL), H_2_O, CH_3_CN, and DCM were used (entries 1–2, 5, and 8). Interestingly, the addition of a base dramatically increased the yield (entries 9–11). The addition of 1 eq. of bases such as *t*-BuOK, K_2_CO_3_, or CS_2_CO_3_ afforded azobenzene in higher yields (72–99%); in particular, K_2_CO_3_ provided a quantitative yield with 100% selectivity and conversion of hydrazobenzene (entry 10). To verify the role of air in this reaction, when the reaction was carried out under a nitrogen atmosphere, the yield of azobenzene decreased to 56% (entry 12). This result revealed the importance of air as oxygen from the air is responsible for aerobic oxidation. In this context, Gozin and coworkers previously reported the oxidative dehydrogenation reaction using H_2_O_2_ as an external oxidant.^19^

**Table 3:** Oxidation of hydrazobenzene over Ni–Fe@N-doped-C under various solvents and basic additives^a^

Entry	Solvent	Base [eq.]	Recovered hydrazobenzene [%]	Azobenzene yield [%]
1	GVL	None	51	35
2	H_2_O	None	60	26
3	PEG-400	None	41	50
4	Ethylene glycol	None	38	49
5	CH_3_CN	None	55	39
6	THF	None	43	49
7	*t*-BuOH	None	45	42
8	DCM	None	68	15
9	EtOH	*t*-BuOK (1)	21	72
10	EtOH	K_2_CO_3_ (1)	00	99
11	EtOH	CS_2_CO_3_ (1)	11	81
12^b^	EtOH	K_2_CO_3_ (1)	38	56

a Reaction conditions: 1 mmol hydrazobenzene, 5 wt% Ni–Fe@N-doped-C, 3 mL solvent, base, 30 °C for 12 h under open air.

b Under N_2_ atmosphere.

Moreover, the oxidative dehydrogenation reactions of different hydrazobenzenes were further investigated with 5 wt% Ni–Fe@N-doped-C catalyst under optimal conditions and the obtained data are listed in Table 4. It was found that all the electron-donating or electron-withdrawing group-bearing hydrazobenzenes could be efficiently transformed to azobenzenes with excellent selectivity and yields (entries 1–6).

**Table 4:** Oxidation of various hydrazobenzenes over the Ni–Fe@N-doped-C catalyst

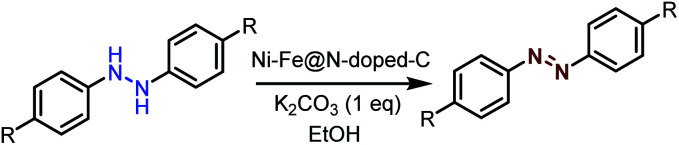			
Entry	Starting	Product	Yield [%]
1	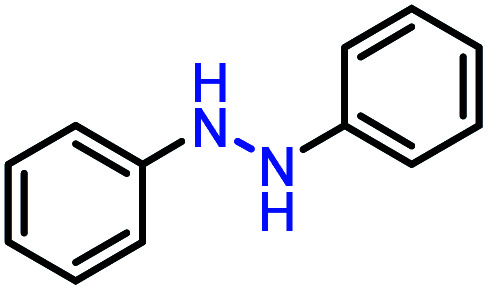	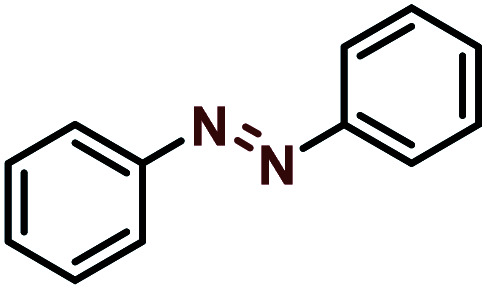	99
2	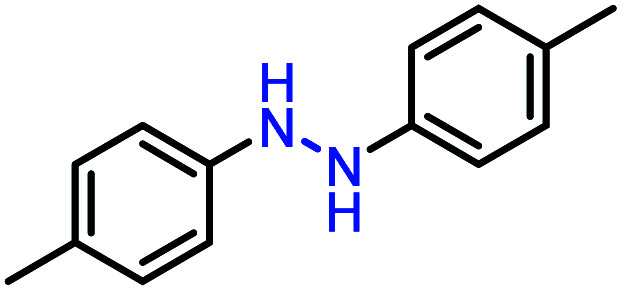	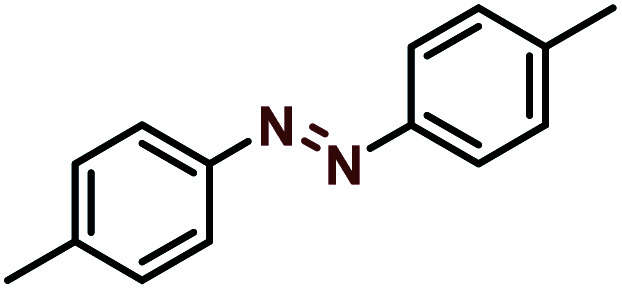	98
3	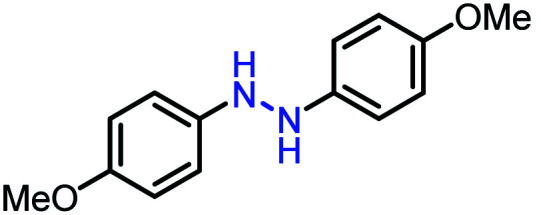	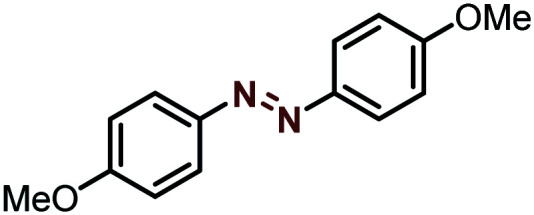	99
4	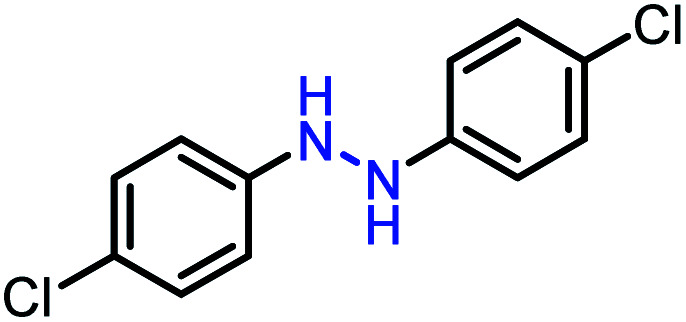	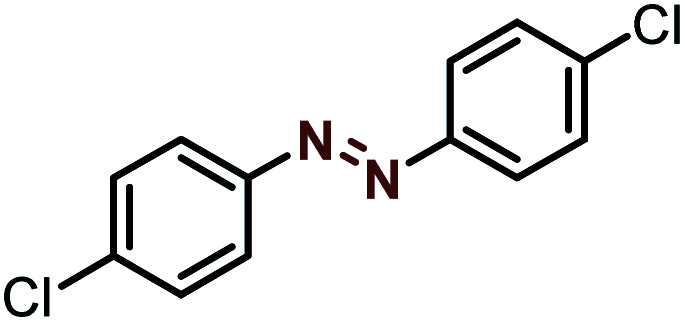	91
5	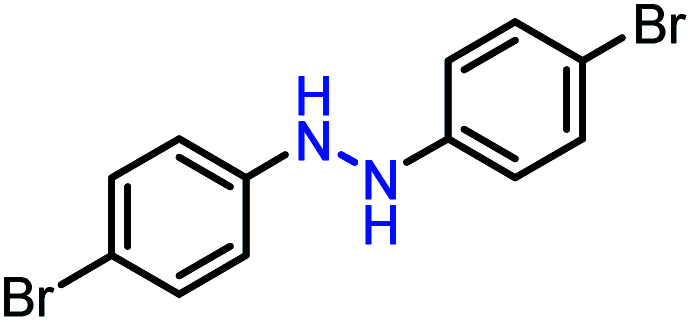	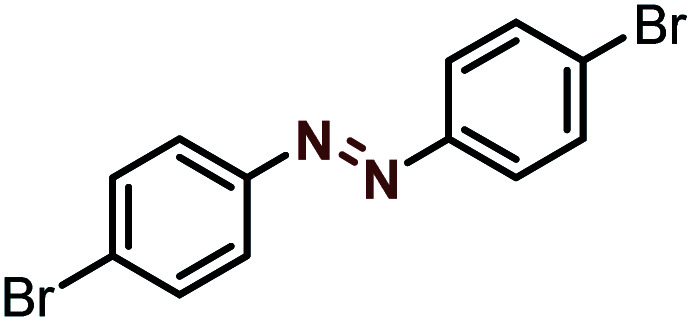	92
6	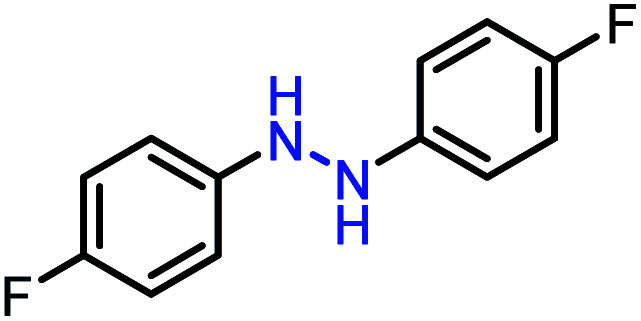	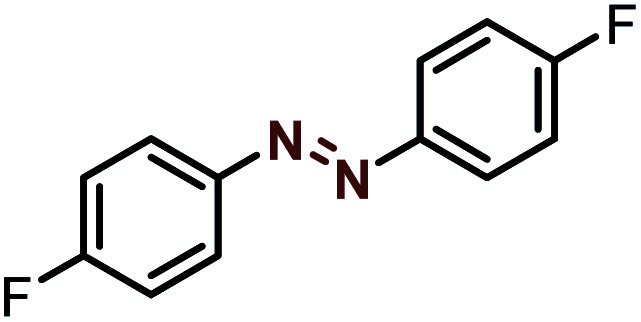	90

**Scheme 2:**
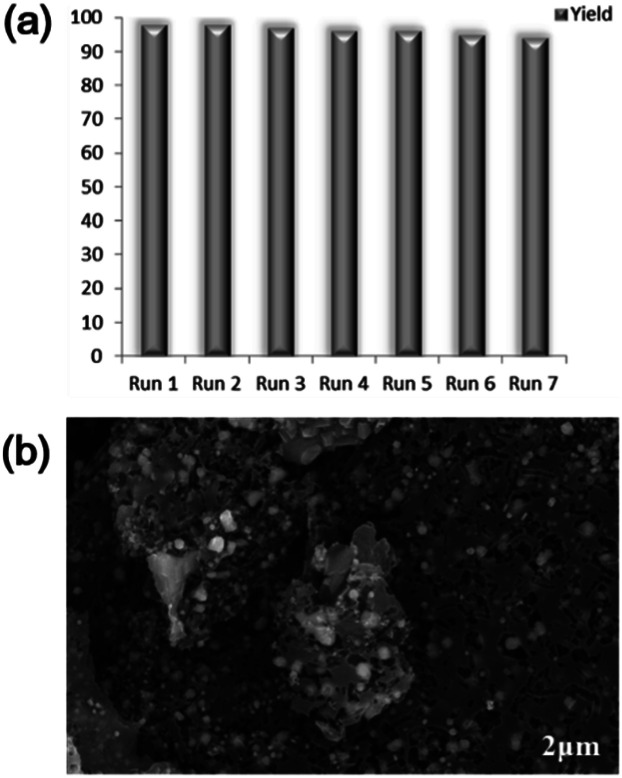
(a) Reusability of Ni–Fe@N-doped-C; (b) SEM image of reused Ni–Fe@N-doped-C after the 7th run.

To demonstrate the large-scale applicability, we carried out a gram-scale reaction of hydrazobenzene and obtained 1.66 g of azobenzene in a 91% yield (for details, see ESI, Fig. S4†). The recycling experiments of the Ni–Fe@N-doped-C catalyst were also studied. The reaction was performed under optimized reaction conditions (1 mmol scale). After completing the reaction, the catalyst was magnetically separated from the reaction mixture (Fig. S4†) and washed with water and acetone 3–4 times. The collected catalyst was further dried at 60 °C for 24 h. The spent catalyst was used for the next cycle under optimized reaction conditions. The catalyst could be recycled in seven successive runs, sustaining the selectivity of the reactant and the yield of azobenzene. Only a little decrease in the conversion of hydrazobenzene from 99% to 95% was observed upon the seventh reuse (Scheme 2a). After the seventh recycle, the SEM image of Ni–Fe@N-doped-C shows no significant change in the morphology and structure (Scheme 2b). The metal concentration in the reused Ni–Fe@N-doped-C catalyst was found to be 10.29 wt% for Ni and 14.62 wt% for Fe by ICP-AES analysis.

The efficiency of the synthesized catalyst was studied by comparing it with previously reported catalysts. In this context, Drug *et al.* reported the oxidation reaction of hydrazobenzene by using a TiCl_3_/HBr catalytic system (Table 5, entry 1),^19^ whereas Sahoo *et al.* reported the dual transition-metal-catalysed synthesis of azobenzene (entry 2).^20^ In addition, the report by Du *et al.* showed the electrochemical dehydrogenation of hydrazine compounds using a Pt electrode (entry 3).^42^ In another report, reduced graphene oxide was utilized for the dehydrogenation reaction (entry 4).^43^ However, these methods have certain limitations, such as toxicity, non-reusability, the use of noble metals or additives, and high catalyst loading. In this study, the catalytic activity of the reusable Ni–Fe@N-doped-C catalyst was proven to be better than that of previously reported catalysts.

**Table 5:** Comparative study

Entry	Catalyst	Yield [%]	Ref.
1	TiCl_3_/HBr	95	19
2	[Ru(bpy)_3_]^2+^ + Co(dmgH)_2_(py)Cl	95	20
3	Pt electrode	97	42
4	rGO	98	43
5	Ni–Fe@N-doped-C	99	Present work

### Reaction mechanism

**Scheme 3:**
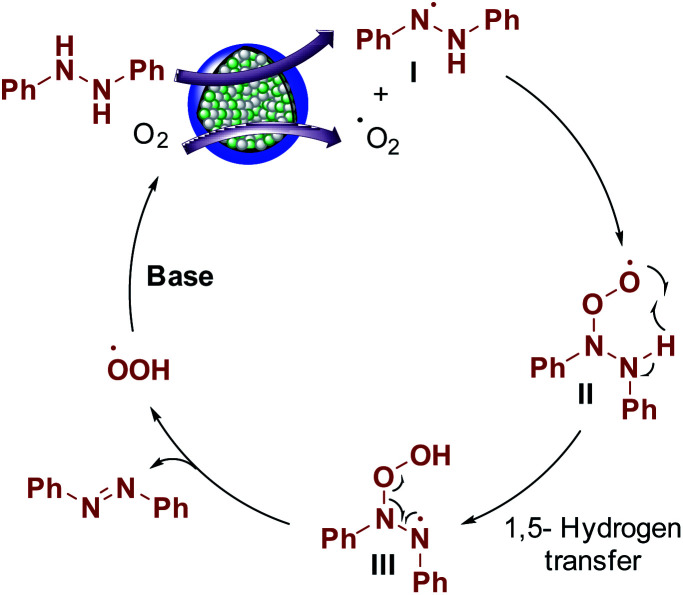
Reaction mechanism.

Aerobic oxidation reactions involve the generation of radicals over carbon catalysts.^44,45^ Wen, Su, and coworkers have reported alkene radical generation over a carbon catalyst under aerobic conditions.^46^ According to our observations and previous reports, we assumed that the reaction mechanism proceeded through the formation of radicals and proposed the possible reaction mechanism (Scheme 3). Hydrazobenzene was activated on the graphitic layer surface to form nitrogen radicals (I), and O_2_ was activated on the carbon adjacent to graphitic N. The electron transfer from adjacent carbon atoms to O_2_ to form O_2_ radical was possible due to graphitic N atoms and metallic species.^44,47^ Then, these two radicals interacted with each other to form a peroxy radical intermediate II and the subsequent radical transfer on to the adjacent nitrogen generated another radical intermediate III. Finally, deoxygenation occurred to form azobenzene as the final product with the elimination of a peroxy radical. Finally, in the presence of a base, the peroxy radical was converted to O_2_ to complete the catalytic cycle. In general, the graphitic layered structure, N-species, bimetals, and base are equally critical for this transformation.

## Conclusions

Environmentally friendly and sustainable bimetallic Ni–M (M = Co/Fe/Cu) nanocomposites encased by N-doped mesoporous carbons using naturally renewable bio-waste (chitosan) were introduced. The synthesized catalysts were composed of mixed phases of metallic, metal oxide, and N-doped carbon layers. The Ni–Fe@N-doped-C catalyst possessed a large surface areawith mesoporous structure and exhibited good catalytic activity in the dehydrogenation of hydrazobenzene reactions compared with the conventional catalysts. When Ni–Fe@N-doped-C was the paragon catalyst, a high yield of azobenzene (99%) could be obtained in EtOH as a solvent. The catalyst was reused for seven cycles without a prominent loss of catalytic activity. The research hence emphasizes a new aspect for the synthesis of non-noble metal-based N-doped carbon catalysts and their application in the large-scale dehydrogenation of hydrazobenzene.

## Conflicts of interest

There are no conflicts to declare.

## Supplementary Material

RA-010-C9RA08146A-s001
