# Supplementary material for: Chitosan-derived N-doped carbon catalysts with a metallic core for the oxidative dehydrogenation of NH–NH bonds
Source: RSC Adv. 2020 Jan 2;10(1):474–81. doi: 10.1039/c9ra08146a (PMC9047065; doi:10.1039/c9ra08146a)
Supplement: RA-010-C9RA08146A-s001 [file RA-010-C9RA08146A-s001.pdf]

Electronic Supplementary Material (ESI) for RSC Advances.
This journal is © The Royal Society of Chemistry 2019

Chitosan-derived *N*-doped carbon catalysts with metallic core for oxidative dehydrogenation of NH-NH bonds

Priyanka Raju Thombal, Raju S. Thombal, and Sung Soo Han*

School of Chemical Engineering, Yeungnam University, Gyeongsan 712-749, Republic of Korea

Supplementary Information

Table of Contents

Materials S2

Procedures:

i) General procedure for the synthesis of catalysts S2

ii) General procedure for the synthesis of azobenzene S3

Additional characterization of catalysts:

i) EDS S3

ii) XRD S4

iii) N_2 adsorption/desorption isotherms & BJH pore distributions S5

Additional experiments:

i) Large scale reaction S5

ii) Reusability test S6

References S6

Materials:

Chitosan, Hydrazobenzene, $NiCl_2.6H_2O$, $CoCl_2.2H_2O$, $FeCl_3$, $CuCl_2.2H_2O$, Ammonia water etc. were purchased from the standard chemical suppliers such as Sigma Aldrich and Duksan. All the chemicals were reagent grade and used as received without further purification.

Procedures:

i) General procedure for the synthesis of catalysts:

The chitosan supported bimetallic materials were synthesized according to the following typical procedure: In a 500mL round-bottomed flask provided with a magnetic stir bar, 1.5 gm of chitosan was suspended into 100 mL of water. 250 mg of each $NiCl_2.6H_2O$ and metal precursors (Co/Fe/Cu) were added into above suspension solution. The pH was maintained around 9 by adding 25% aqueous ammonia solution and the mixture was stirred continuously for 24 h. Hereafter, the obtained solid was repeatedly washed with water to remove excess impurities and reactants. The solid was then separated by centrifugation process (500 rpm, 5 min) and dried in vacuum at 60 ^{o}C overnight. Later, the dried sample was transferred into a tubular furnace for pyrolysis. The furnace then flushed with argon and sample was heated to 700 ^{o}C temperature with a temperature gradient of 25 °C/min and the same temperature was held for 2h. After that, the furnace was cooled down to rt. Argon was purged through the furnace constantly during the whole process. The obtained black material was washed with water and ethanol to remove the impurities. The catalysts named as Ni-M@N-doped-C (M=Co/Fe/Cu) were obtained after drying the samples at 60 ^{o}C in an oven and stored in a screw-capped vial at room temperature. For the preparation of monometallic catalysts, same procedure was followed using single metal precursors.

Yields obtained after pyrolysis:

i) Ni-Co@N-doped-C: 31 wt%
ii) Ni-Fe@N-doped-C: 27 wt%
iii) Ni-Cu@N-doped-C: 45 wt%

The product yield is determined by the following equation:

Product yield (%) = amount obtained after pyrolysis/amount injected for pyrolysis X 100.

ii) **General procedure for the synthesis of azobenzene:**

Typically, a calculated amount of hydrazobenzene (1 mmol), Ni-Fe@N-doped-C (5 wt%) in ethanol (3 mL) were placed in a 10 mL glass vial, and K_2CO_3 (1 mmol) was added to the mixture under air atmospheric with a magnetic stirring to initiate the reaction at 30 °C for 12 h. After the reaction was completed, the catalyst was separated by external magnet and crude mass was subjected for column chromatography. The obtained product was characterized over NMR spectroscopy and observed data found consistent with the literature report[1]. After each cycle, the catalyst was isolated from the solution by external magnet, washed three times with water and acetone, dried under vacuum to remove the residual solvent and then reused for another reaction cycle.

Additional characterization of catalysts:

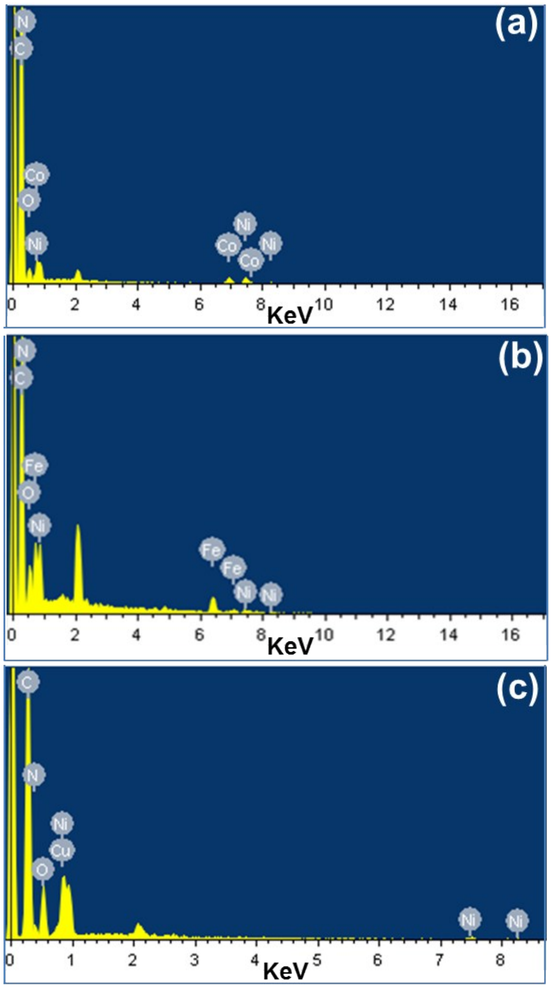

Figure S1. EDS analyses of (a) Ni-Co@N-doped-C, (b) Ni-Fe@N-doped-C, and (c) Ni-Cu@N-doped-C catalysts.

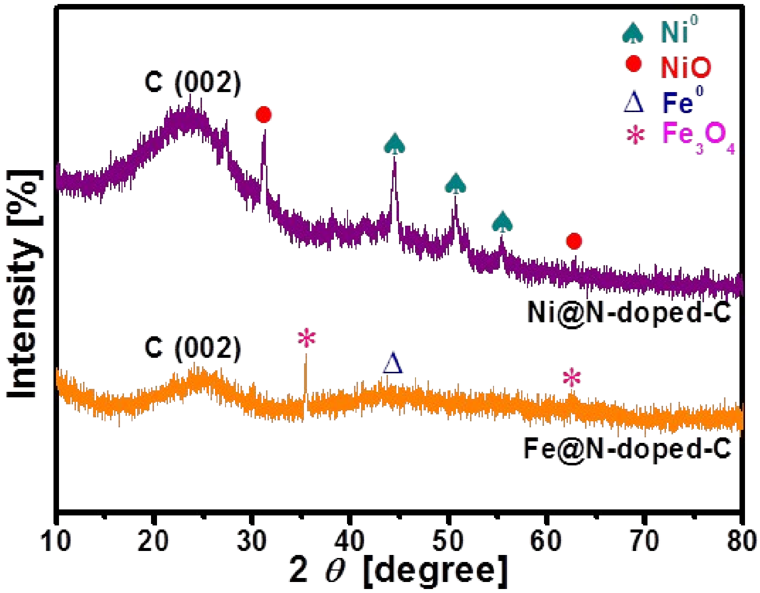

Figure S2. Wide-angle XRD patterns of monometallic catalysts

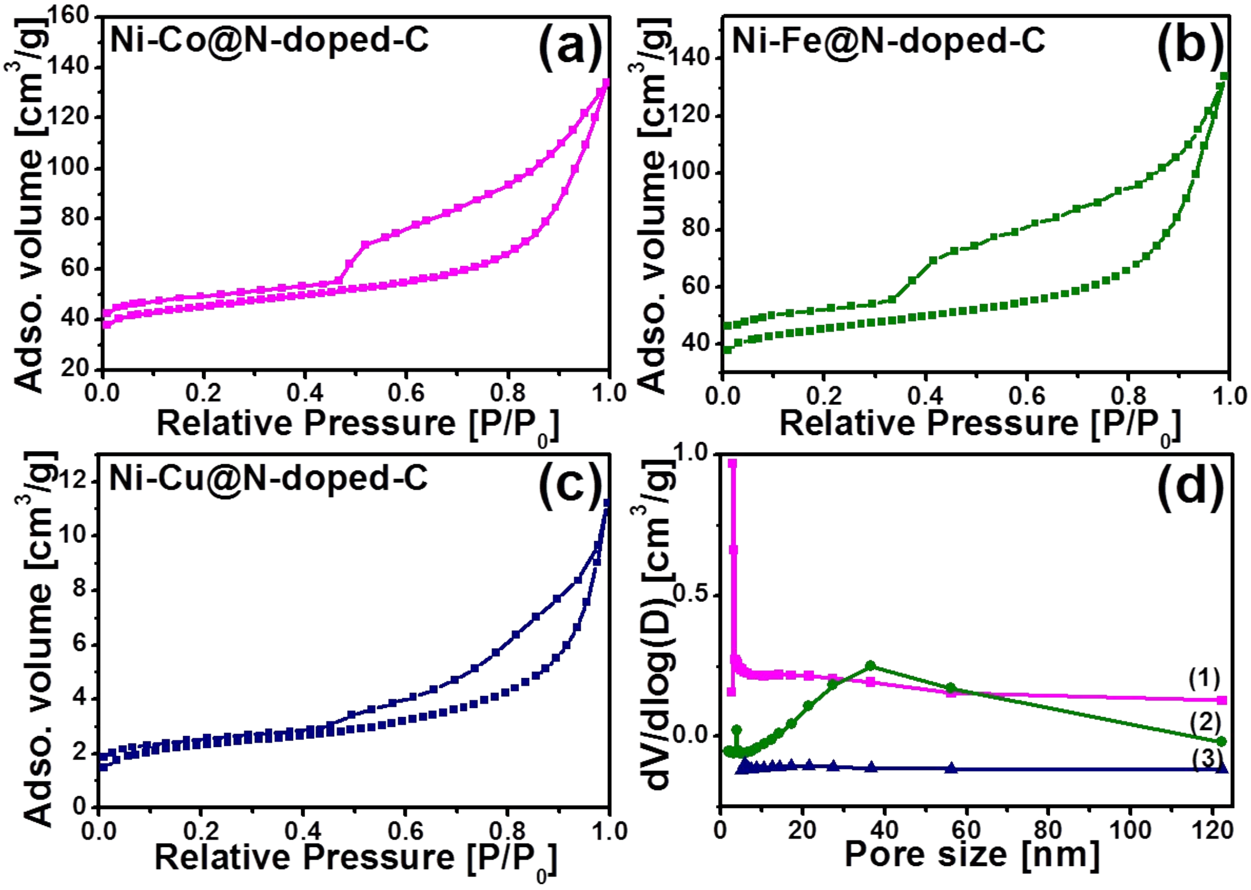

Figure S3. N_2 adsorption/desorption isotherm of (a) Ni-Co@N-doped-C, (b) Ni-Fe@N-doped-C, (c)Ni-Cu@N-doped-C and (d) BJH pore distributions of (1) Ni-Co@N-doped-C, (2) Ni-Fe@N-doped-C, and (3) Ni-Cu@N-doped-C catalysts respectively.

Additional experiments

Large scale reaction:

The mixture of hydrazobenzene (1.84g, 10 mmol), K_2CO_3 (1.38g, 10 mmol), Ni-Fe@N-doped-C (5 wt%) in ethanol (21 mL) were placed in a 50 mL round bottle flask, and mixture stirred under air atmospheric at 30 °C for 12 h. After the reaction was completed, the catalyst was separated

by external magnet and crude mass was subjected for column chromatography. The yield of obtained azobenzene was observed in 91% (1.66 g).

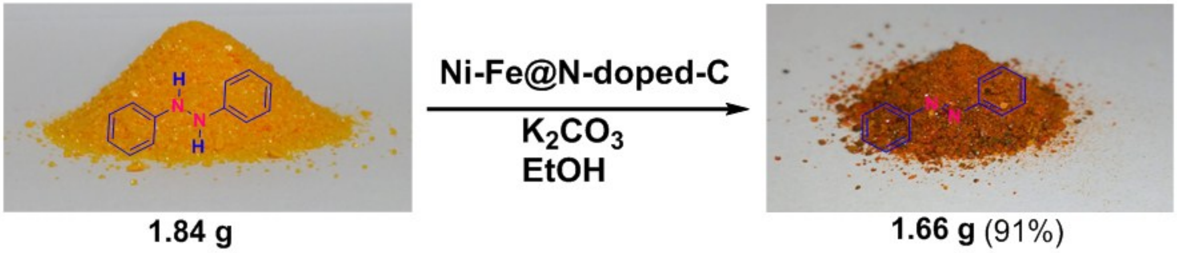

Figure S4. Gram-scale synthesis of azobenzene over Ni-Fe@N-doped-C.

Reusability test

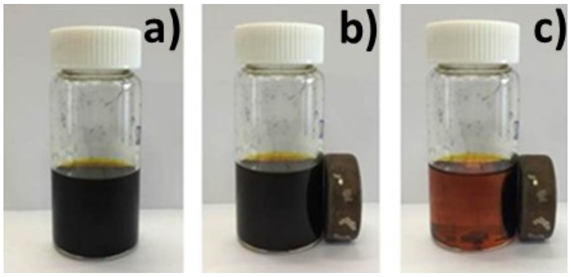

Figure S4. Separation of the Ni-Fe@N-doped-C catalyst (a,b) after reaction and (c) separation by using permanent magnet.

Reference

1 E. Drug, and M. Gozin, *J. Am. Chem. Soc.*, 2007, **129**, 13784-13785.
